# Pan‐cancer analysis of SERPINE1 with a concentration on immune therapeutic and prognostic in gastric cancer

**DOI:** 10.1111/jcmm.18579

**Published:** 2024-07-31

**Authors:** Yuming Ju, Zeshen Wang, Qiancheng Wang, Shiyang Jin, Pengcheng Sun, Yuzhe Wei, Guanyu Zhu, Kuan Wang

**Affiliations:** ^1^ Department of Gastrointestinal Surgery Harbin Medical University Cancer Hospital Harbin China

**Keywords:** bioinformatics, immune infiltrating, pan‐cancer analysis, SERPINE1

## Abstract

The serine protease inhibitor clade E member 1 (SERPINE1) is a key modulator of the plasminogen/plasminase system and has been demonstrated to promote tumor progression and metastasis in various tumours. However, although much literature has explored the cancer‐promoting mechanism of SERPINE1, the pan‐cancer analyses of its predictive value and immune response remain unexplored. The differential expression, and survival analysis of SERPINE1 expression in multiple cancers were analysed using The Cancer Genome Atlas and Genotype‐Tissue Expression database. Kaplan–Meier (K–M) plotter and survival data analysis were used to analyze the prognostic value of SERPINE1 expression, including overall survival (OS), disease‐specific survival, disease‐free interval and progression‐free interval and investigated the relationship of SERPINE1 expression with microsatellite instability. We further analysed the correlation between the expression of SERPINE1 and immune infiltration. The Kyoto Encyclopaedia of Genes and Genomes pathway was used for enrichment analysis, and the Gene Set Enrichment Analysis (GSEA) database was used to perform pathway analysis. Finally, in vitro experiments demonstrated that knockdown or overexpression of SERPINE1 could alter the proliferation and migration of gastric cancer (GC) cells. The results indicated that SERPINE1 expression levels different significantly between cancer and normal tissues, meanwhile, it was highly expressed in various cancers. By analysing online data, it has been observed that the gene SERPINE1 exhibits heightened expression levels across a variety of human cancers, significantly impacting patient survival rates. Notably, the presence of SERPINE1 was strongly associated with decrease OS and disease‐free survival in individuals diagnosed with GC. Furthermore, an observed link indicates that higher levels of SERPINE expression are associated with increased infiltration of immune cells in GC. Finally, in vitro experiments showed that knockdown or overexpression of SERPINE1 inhibited the growth, and migration, of GC cells. SERPINE1expression potentially represents a novel prognostic biomarker due to its significant association with immune cell infiltration in GC. This study shows that SERPINE1 is an oncogene that participates in regulating the immune infiltration and affecting the prognosis of patients in multiple cancers, especially in GC. These findings underscore the importance of further investigating the role of SERPINE1 in cancer progression and offer a promising direction for the development of new therapeutic strategies.

## 1 INTRODUCTION

Cancer represents a significant public health challenge globally and stands as the leading cause of mortality in the world.^1^ Despite advancements in surgical and medical technologies, cancer treatment continues to pose a formidable challenge. In recent years, the limitations of surgery and chemotherapy in fully addressing cancer patient needs have led to the emergence of immunotherapy as a promising new avenue for cancer treatment.^2^, ^3^ Consequently, the search for valuable diagnostic and prognostic biomarkers has become increasingly important in enhancing cancer patient care. Serine protease inhibitor clade E member 1 (SERPINE1) belongs to the serine protease inhibitor family and is the primary inhibitor of both uridylyl phosphate adenosine (uPA) and tissue plasminogen activator (tPA)^4^, ^5^ The balance between the coagulation system and the fibrinolytic process in the blood is critically maintained. Upon activation by tPA, plasminogen transforms into the active enzyme plasmin, which then breaks down the insoluble fibrin meshwork into soluble fragments, facilitating the dissolution and absorption of blood clots. The u‐PA system, integral to the plasminogen activation process, plays a key role in cell migration and tissue remodelling across various biological processes.^6^ SERPINE1 is composed of a single‐chain, non‐glycosylated polypeptide chain of 400 amino acids, with a molecular weight of 50 kDa.^7^ Advances in high‐throughput sequencing techniques have revealed abnormal SERPINE1 expression in several cancers, including Colon cancer (CRC),^8^, ^9^, ^10^ GC,^11^ Pancreatic cancer (PAAD),^12^, ^13^, ^14^ Hepatocellular Carcinoma (HCC).^15^, ^16^ In this study, we investigated the expression of SERPINE1 in various cancers, as well as its prognostic significance and immunological role, using multiple public databases. Furthermore, the study comprehensively analysed the potential association of SERPINE1 with clinical characteristics, tumour mutation burden (TMB), microsatellite instability (MSI), and immune infiltration. We discovered that the expression of SERPINE1 was significantly associated with survival prognosis in GC and exhibited high diagnostic value for GC. More importantly, through experimental validation, we demonstrated that SERPINE1 promotes cell proliferation. The findings of this study suggest that SERPINE1 serves as a potential prognostic biomarker and is closely associated with immune cell infiltration in various tumours, especially in GC.

## 2 MATERIALS AND METHODS

### 2.1 Data collection and expression analysis

We downloaded RNA sequencing data for 33 types of cancer from The Cancer Genome Atlas (TCGA) database, provided in TPM format and acquired corresponding clinical information. Additionally, we obtained expression data for normal tissues from the (Genotype‐Tissue Expression) GTEx database to facilitate comparisons with cancer samples. This control group enables us to identify abnormal changes in expression levels within cancers. Before conducting further analysis, we applied a log2(TPM + 1) transformation to all transcriptome data to minimize skewness and enhance the robustness of statistical analyses. To delve into the expression of the SERPINE1 gene across of cancer, we utilized the Gene Expression Profiling Interactive Analysis (GEPIA) tool.

### 2.2 Analysis of survival

Numerous genes that are highly expressed in cancer tissues can affect patient prognosis. We hypothesized that SERPINE1 influences patient survival. Patients were divided into high and low expression groups based on the median mRNA expression of SERPINE1 across all cancers. Univariate Cox regression analysis focused on OS, disease‐specific survival (DSS), disease‐free interval (DFI), progressionfree interval (PFI). Forest plots were generated using the ‘survival’ and ‘forestplot’ R packages. Additionally, K‐M survival curves were performed to assess the association between SERPINE1 expression and prognosis.

### 2.3 Immune correlation analysis

The microenvironment is essential for the survival and progression of tumour cells. We used the ESTIMATE algorithm to calculate the stromal scores and immune scores in each tumour based on the R package ‘ESTIMATE’. Additionally, the relative scores for 24 immune cells in 33 cancers were assessed by CIBERSORT, which can predict the phenotypes of immune cells with R packages ‘limma’ and ‘CIBERSORT’. The correlation data between SERPINE1 expression and six major immune cell types of Tumour‐infiltrating immune cells (TIICs)—B cell, CD4+ T cell, CD8+ T cell, neutrophil, macrophage and dendritic cell—were analysed. Results were presented as Immune Scores and Stromal Scores. Higher Immune Scores or Stromal Scores indicated a higher immune or stromal component ratio, and an increase in the respective score was associated with a larger ratio.

### 2.4 Relation of SERPINE1 MSI and TMB analysis

Tumour mutation burden and MSI are quantifiable immune‐response biomarkers defined as the total number of mutations in tumour samples and are promising biomarkers for predicting immune response. We obtained corresponding clinical information and RNA‐sequencing data for SERPINE1 from the TCGA dataset to explore its impact. The analyses of the correlations between SERPINE1 expression and both MSI and TMB were conducted using R software version 4.0.3, employing Spearman's correlation method.

### 2.5 Gene set enrichment analysis identifies biological pathways

To explore the potential biological and molecular mechanisms of SERPINE1 in GC, this study utilized Gene Set Enrichment Analysis (GSEA) to analyse the differences in predefined gene sets between two comparison groups using the ‘clusterProfiler’ and ‘enrichplot’ packages in R software. Additionally, Kyoto Encyclopaedia of Genes and Genomes (KEGG) pathway enrichment analysis was performed on genes related to SERPINE1 expression to identify significantly associated biological processes and signalling pathways. This provides important insights for further understanding the role of SERPINE1 in disease.

### 2.6 Cell culture

GC cell lines (HGC‐27 and BGC‐823) were provided by Cell Bank of the Chinese Academy of Science, Shanghai. HGC‐27 and BGC‐823 were grown in RPMI 1640 medium (supplied by Gibco, United States) enriched with 10% foetal bovine serum. These cells were maintained at a constant temperature of 37°C within a humidified incubator, ensuring an atmosphere containing 5% CO2. Small interfering RNAs (siRNAs) designed to silence the target genes, along with siRNAs serving as negative controls, were produced by Hanbio Biotechnology Co. Ltd (Shanghai, China). Meanwhile, plasmids for overexpressing the target genes and their respective empty vectors were generated by RiboBio (Guangzhou, China).

### 2.7 Western blotting

Proteins were isolated from either cell lines or tissue samples using ice‐cold RIPA buffer (obtained from Beyotime Institute of Biotechnology, Shanghai, China), and protein concentrations were measured using a BCA Protein Assay Kit (also from Beyotime Institute of Biotechnology, Shanghai, China). Subsequently, a solution with a concentration of 2 mg/mL was prepared using 4X loading buffer (sourced from GenScript Tech, Nanjing) and samples were denatured by heating. Proteins were then subjected to electrophoresis on a 4–20% SDS‐polyacrylamide gel and electrotransferred onto PVDF membranes (Millipore, United States). The membranes were blocked with 5% non‐fat milk for 1 h at ambient temperature and incubated with primary antibodies at 4°C overnight. Following multiple washes, secondary antibodies were applied to the membranes for 1 h at ambient temperature the next day. The immunoreactive bands were visualized using enhanced chemiluminescence (ECL) detection reagents (Thermo Fisher Scientific™), and the intensity of the protein bands was analysed with ImageJ software.

### 2.8 Wound healing assay

Well‐growing cells were seeded into a 6‐well plate, ensuring that 37°C and straight lines were drawn at the bottom of each well. A micropipette equipped with a 200 μL tip was used, and with the aid of a ruler, the tip was held perpendicular to the plate and smoothly slid from top to bottom to guarantee even cell distribution. Using the micropipette, 1 mL of PBS was gently added to each well, followed by washing with PBS 2–3 times and then serum‐free medium was introduced. The plate was then placed back into the incubator for further cultivation. At predetermined time points of 0, 24 and 48 h, the cells were observed under a microscope and photographed. The experiment was repeated multiple times, and cell migration distances were calculated from the photographs and subjected to statistical analysis.

### 2.9 Transwell assays

In the lower chamber of the Transwell apparatus, 600 μL of complete culture medium was added, while the upper chamber was filled with 200 μL of serum‐free medium, into which a cell density of 3.5 × 10^4^ cells was seeded. The Transwell was then placed in a 37°C incubator for a period ranging from 24 to 48 h. The Transwell was washed 2–3 times with PBS, followed by cell fixation with 4% formaldehyde solution at room temperature for 30 min. The fixative was removed 2–3 times, after which cells were stained with a 0.5% crystal violet solution for 30 min. Post‐staining, the cells were rinsed, and a cell scraper was used to remove cells from the interior of the upper chamber. The cells within the Transwell were magnified at an appropriate scale, and then 5 random fields of view were photographed under a microscope.

### 2.10 Cell proliferation assay

The Cell Counting Kit‐8 (CCK‐8) method was utilized to assess cell proliferation rates. Cells (2000 cells/well) were distributed into 96‐well plates. Then, 10 μL of CCK‐8 solution was introduced into each well. The plates were incubated for 2 h at 37°C, and the optical density was recorded at 450 nm. This procedure was performed daily over a 24‐h interval.

### 2.11 Statistical analysis

The Wilcoxon rank‐sum test was used to evaluate differences in gene expression between tumour tissues and normal tissues across various types of tumours. Survival analyses were performed using the Kaplan–Meier method or Cox regression models with log‐rank tests. Correlations between two variables were studied using either the Spearman or Pearson methods. ImageJ software was utilized to analyse results from Western Blot, wound healing, Transwell and CCK‐8 migration assays; statistical processing of data was conducted using SPSS software version 26.0, while GraphPad Prism 9 was used for further statistical analysis and graphical presentation. To ensure the reliability of experimental results, each experiment was performed at least three times. In all conducted data analyses, a *p*‐value of less than 0.05 was considered statistically significant, where *p < 0.05, **p < 0.01, ***p < 0.001 and ****p < 0.0001.

## 3 RESULTS

### 3.1 Differential expression of SERPINE1 in tumours

**FIGURE 1:**
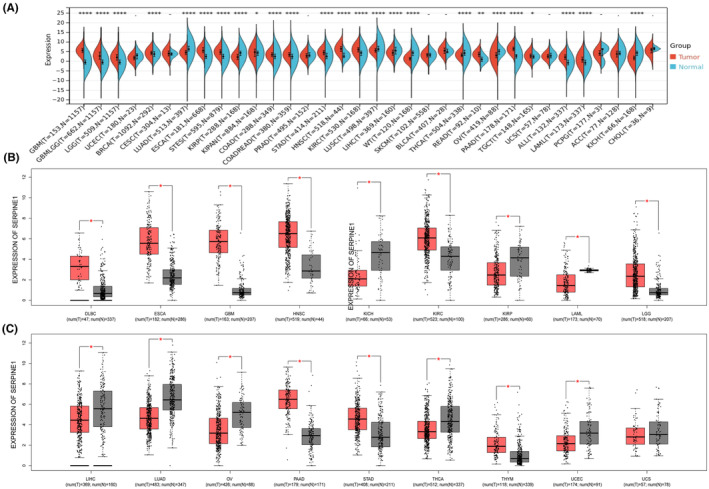
Expression levels of SERPINE1 in human tumours. (A–C) Expression of SERPINE1 in pan‐cancer(*p < 0.05, **p < 0.01, ***p < 0.001, ****p < 0.0001).

We compared the differences in SERPINE1mRNA expression between cancer and normal tissues, and SERPINE1 expression in cancer was significantly different from that in normal tissues except for cancers with no normal tissue data or only a few normal samples (Figure 1A). SERPINE1 is upregulated in 14 cancer types, including Glioblastoma multiforme (GBM), Brain Lower Grade Glioma (LGG), Breast invasive carcinoma (BRCA), Oesophageal carcinoma(ESCA), Stomach and Oesophageal carcinoma (STES), Pan‐kidney cohort (KIPAN), Colon cancer (COAD), Stomach adenocarcinoma, (STAD), Neck squamous cell carcinoma (HNSC), Kidney renal papillary cell carcinoma (KIRC), Rectum adenocarcinoma (READ), Pancreatic adenocarcinoma, (PAAD), Acute Myeloid Leukaemia (ALL). SERPINE1 is associated with Lung adenocarcinoma (LUAD), Kidney renal papillary cell carcinoma (KIRP), Lung squamous cell carcinoma (LUSC), Liver hepatocellular carcinoma (LIHC), Thyroid carcinoma (THCA), Ovarian serous cystadenocarcinoma(OV), The expression levels of Kidney Chromophobe and kidney chromophobe (KICH), were low in 7 tumours. These results suggest that SERPINE1 is abnormally expressed in many types of cancer, including GC. In addition, we further explored the differential expression of SERPINE1 using the GEPIA database (Figure 1B,C).

### 3.2 Prognostic significnace of SERPINE1 in Pan‐cancer

**FIGURE 2:**
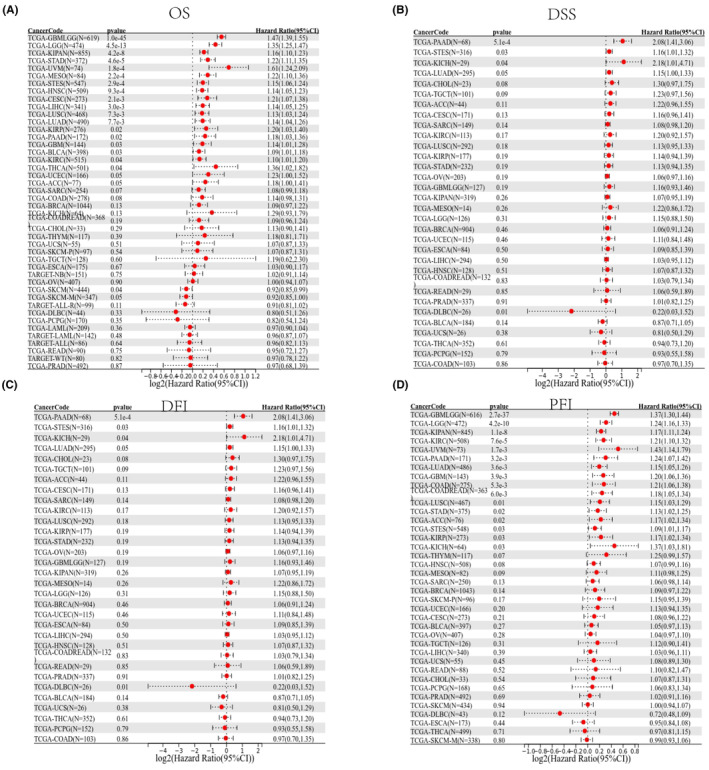
Forest plots showing results of univariate Cox regression analysis for (A) OS, (B) DSS, (C) DFI and (D) PFI.

**FIGURE 3:**
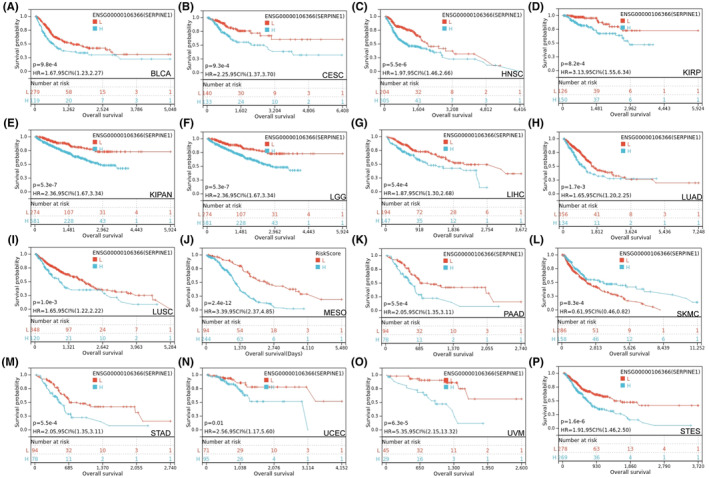
Relationship between SERPINE1 expression and prognosis. (A–P) demonstrated K‐M survival analysis and the relationship between different expression levels of SERPINE1 and overall cancer survival.

To investigate the clinical relevance and prognostic impact of SERPINE1, we generated a forest plot through univariate Cox regression analysis for OS, DSS, DFI, PFI. The OS analysis indicated that high SERPINE1 levels significantly shortened OS in patients with LGG, UCEC, CESC, LUAD, STES, KIRP, STAD, HNSC, GBM, KIRC and LUSC. Notably, patients with SKCM demonstrated improved outcomes, suggesting a complex role of SERPINE1 in this context (Figure 2A). DSS results revealed that higher SERPINE1 expression was linked to poorer outcomes in LGG, UCEC, BRCA, CESC, LUAD, STES, KIRP, COAD, STAD, HNSC, GBM, with it serving as a protective factor only in SKCM (Figure 2B). High SERPINE1 expression was associated with a significant decrease in DFI for PAAD patients (Figure 2C). Furthermore, elevated SERPINE1 levels were significantly correlated with a reduced PFI in LGG, LUAD, STES, KIRP, COAD, STAD, GBM, KIRC and LUSC (Figure 2D). Kaplan–Meier survival analysis also indicated that in patients with LGG, KIPAN, GC, UVM, MESO, STES, UCEC, CESC and MESO (Figure 3A–O), those with higher expression levels of SERPINE1 had shorter survival times, while higher expression in patients with SKCM was associated with a longer overall survival period (Figure 3P). Consequently, the SERPINE1 gene might be intricately linked to the prognosis and advancement of GC.

### 3.3 Relationship between SERPINE1 and tumour immune infifiltration

**FIGURE 4:**
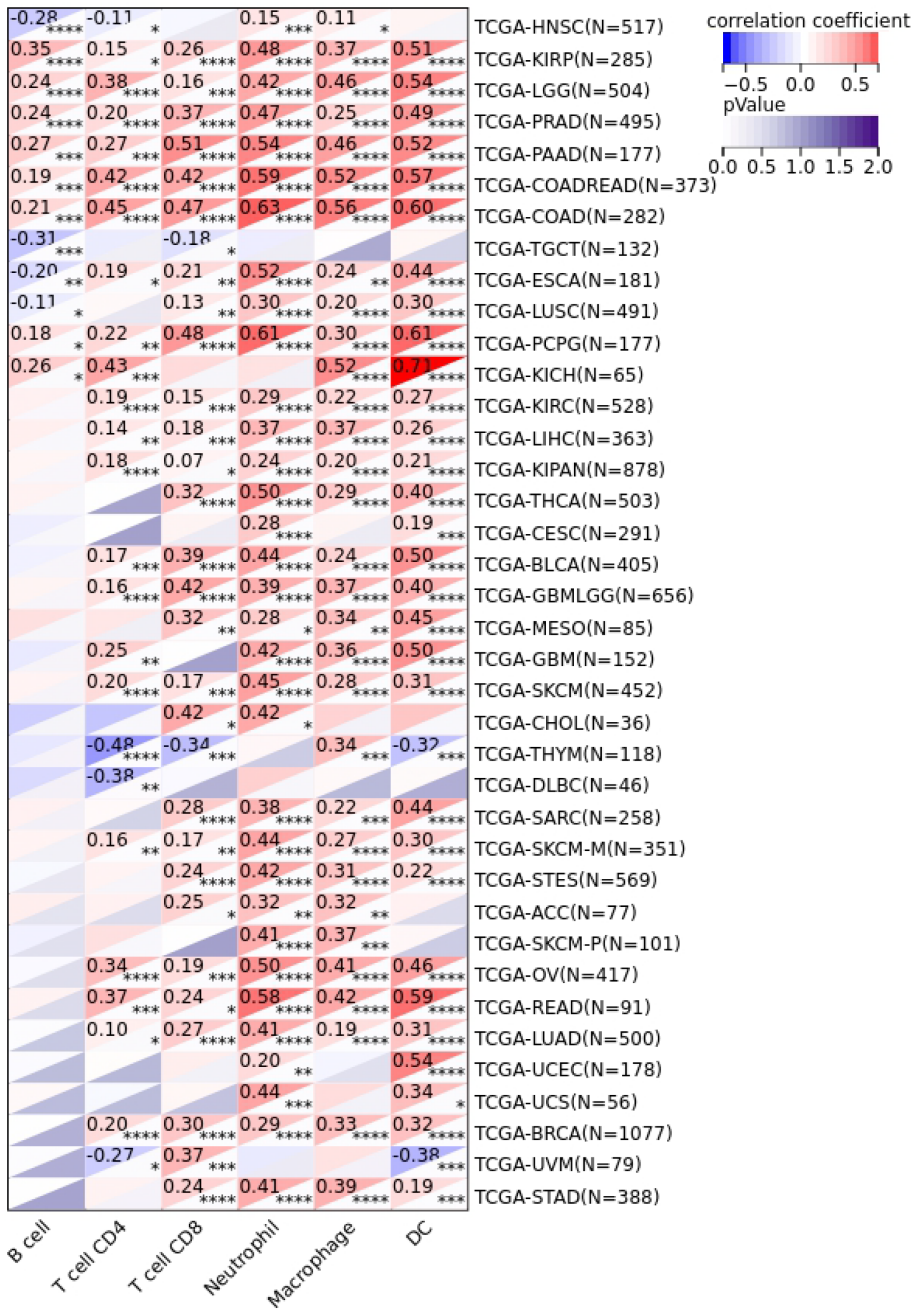
Heat map of correlation between SERPINE1 and tumour immune cell infiltration. *p* Value (*p < 0.05, **p < 0.01, ***p < 0.001, ****p < 0.0001).

**FIGURE 5:**
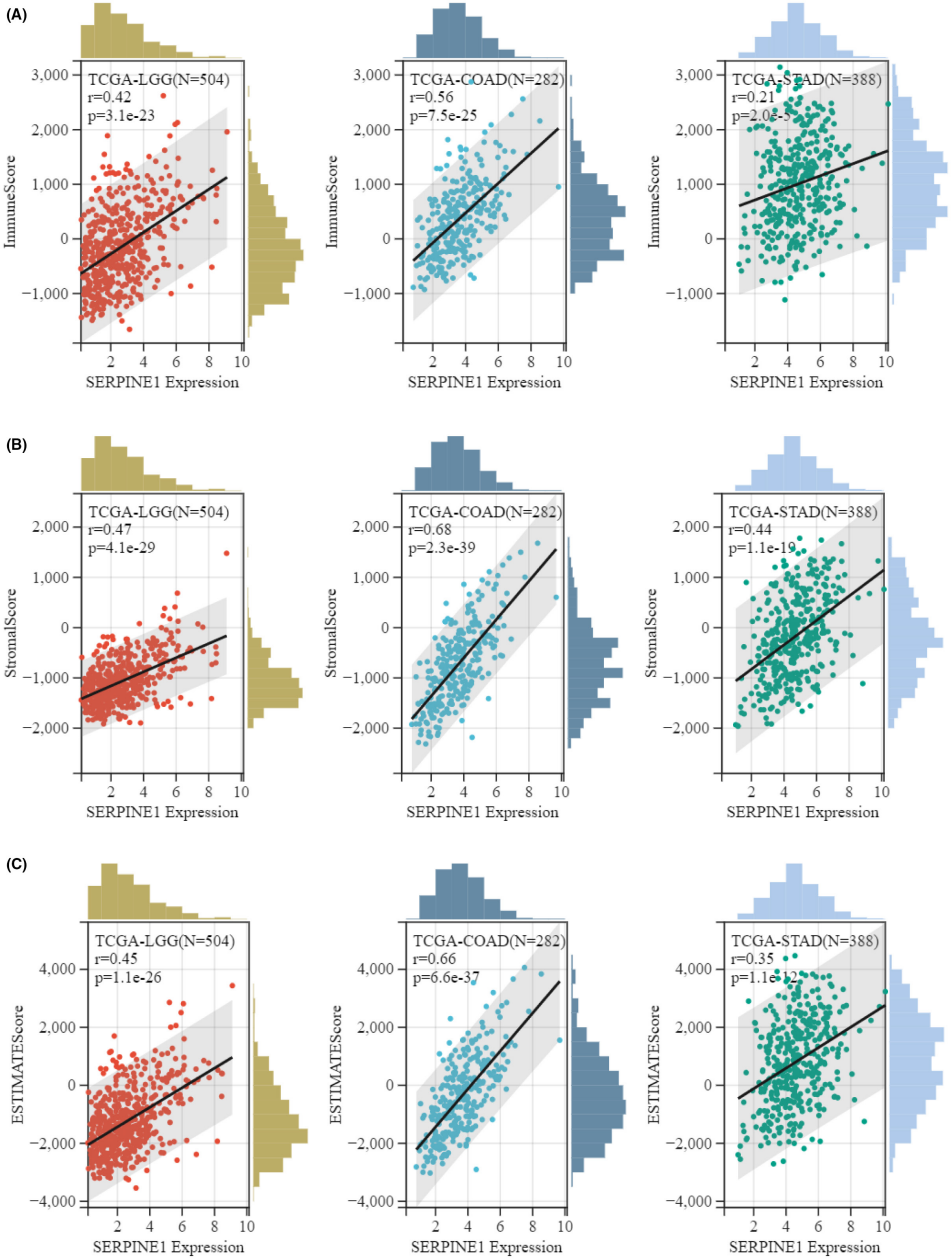
Correlation of Immune Score, Stromal Score and ESTIMATE Score with SERPINE1 Gene Expression in Three Types of Cancer. (A) Immune Score (B) Stromal Score (C) ESTIMATE Score.

Immune cells are implicated in the prognosis of malignant tumours through their influence on the TME. The expression of SERPINE1 is positively correlated with the abundance of infiltrating immune cells. Correlations were observed with CD4+ T cells in 25 different types of cancer, with CD8+ T cells in 25 types, macrophages in 26 types, neutrophils in 19 types and dendritic cells in 22 types (Figure 4A). We further examined the relationship between SERPINE1 expression levels and tumour immune infiltration scores (Immune Score, Stromal Score, ESTIMATE). Select the three cancers with the strongest correlation (LGG, COAD, STAD) for result presentation (Figure 5 A–C). In GC, the expression of SERPINE1 showed a positive correlation with Stromal Score (*r* = 0.44, *p* = 1.1e−19), Immune Score (*r* = 0.21, *p* = 2.0.e−5) and ESTIMATE Score (*r* = 0.35, *p* = 1.1e−12). Our findings demonstrate a correlation between increased infiltration of immune cells and elevated expression of SERPINE1.

### 3.4 Correlation of SERPINE1 expression levels betweend TMB and MSI

**FIGURE 6:**
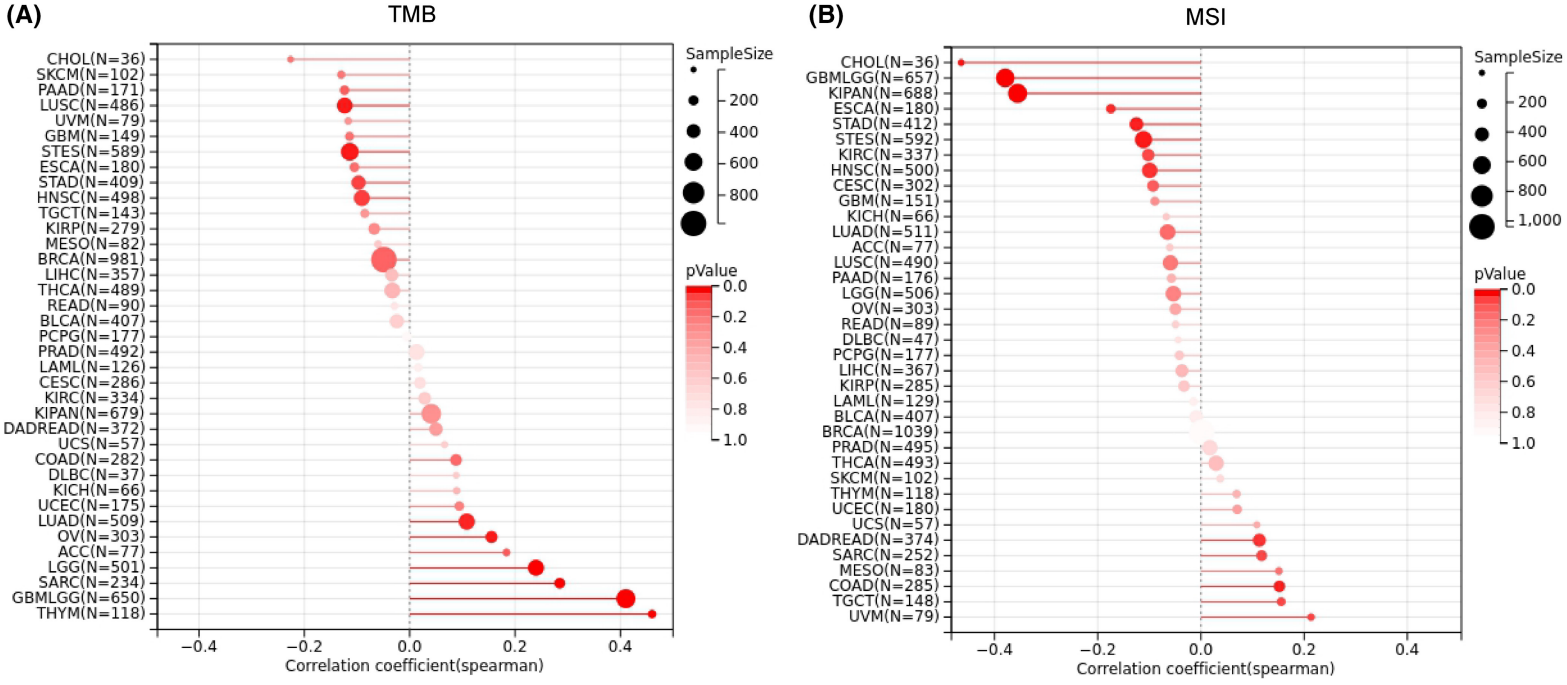
Relationship between the expression level of SERPINE1 and TMB and MSI in pancarcinoma. (A) Relationship between the expression level of SERPINE1 and TMB; (B) Relationship between the expression level of SERPINE1 and MSI.

Tumour mutation burden and MSI are key biomarkers for immunotherapy response. In clinical practice, tumours with high TMB or MSI characteristics often show better responses to immunotherapies like anti‐PD‐1 inhibitors. Therefore, the expression of SERPINE1 was found to be positively correlated with TMB in cancers such as LGG, LUAD, SARC, THYM and OV, while it showed a negative correlation in STES, STAD, HNSC and LUSC (Figure 6A). MSI serves as a prognostic indicator in cancer patients. We analysed the correlation between SERPINE1 expression and MSI in human cancers and found a significant positive correlation in COAD and a negative correlation in ESCA, STES, KIPAN, GC, HNSC and CHOL (Figure 6B). Therefore, TMB or MSI might enhance immune surveillance and the response to immunotherapy. We infer that SERPINE1 may play an indicative role in immunotherapy for several types of cancer.

### 3.5 Functional enrichment analysis of SERPINE1 in human cancer

**FIGURE 7:**
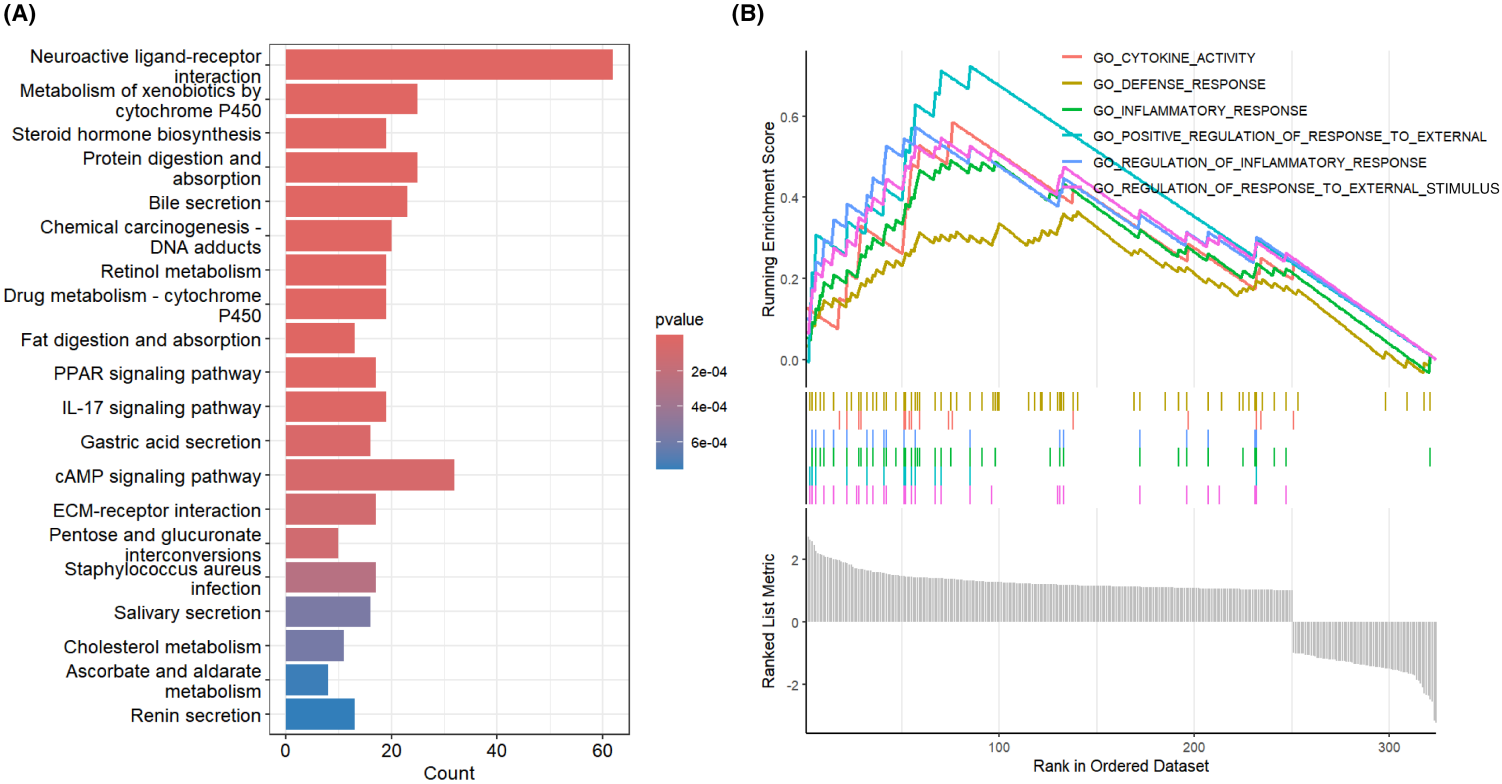
GSEA results of SERPINE1 in gastric cancer. (A) The KEGG pathway is significantly enriched in SERPINE1; (B)The six most highly enriched gene sets in SERPINE1.

To investigate the changes in signalling pathways between high and low SERPINE1 expression groups in GC, GSEA analysis was performed on SERPINE1. KEGG pathway analysis revealed several biological processes significantly associated with SERPINE1 expression, including neuroactive ligand‐receptor interaction, metabolism of xenobiotics by cytochrome P450, steroid hormone biosynthesis, protein digestion and absorption and bile secretion gene sets were notably enriched in the SERPINE1 expression group (Figure 7A). Additionally, our GSEA analysis in the gene set showed that cytokine activity, defence response, inflammatory response, positive regulation of response to external stimulus, regulation of inflammatory response and regulation of response to stimulus were the six most significantly enriched items (Figure 7B).

### 3.6 Expression of SERPINE1 in GC cells

**FIGURE 8:**
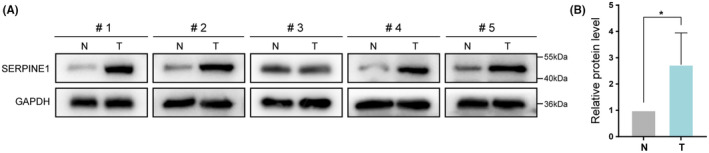
Expression of SERPINE1 in GC cells. (A) The expression level of SERPINE1 in gastric cancer tissues was significantly higher than that in normal tissues. (B) The difference of protein expression in tissues was statistically significant (*p < 0.05).

Based on the results of our analysis, the differential expression of the SERPINE1 gene appears to be closely related to the progression of GC. To gain a deeper understanding of the role and mechanism of SERPINE1 in the development of GC, we utilized the GEPIA database to validate the expression differences of SERPINE1 in GC. The validation results showed that, compared to normal gastric tissues, there was a significant upregulation of SERPINE1 expression in GC patients (Figure 8A). Furthermore, through Western Blot experiments, we confirmed that the protein expression level of SERPINE1 in GC tissues was significantly higher than in adjacent normal tissues, with this difference being statistically significant (Figure 8B).

### 3.7 SERPINE1 promoted the migration of GC cells

**FIGURE 9:**
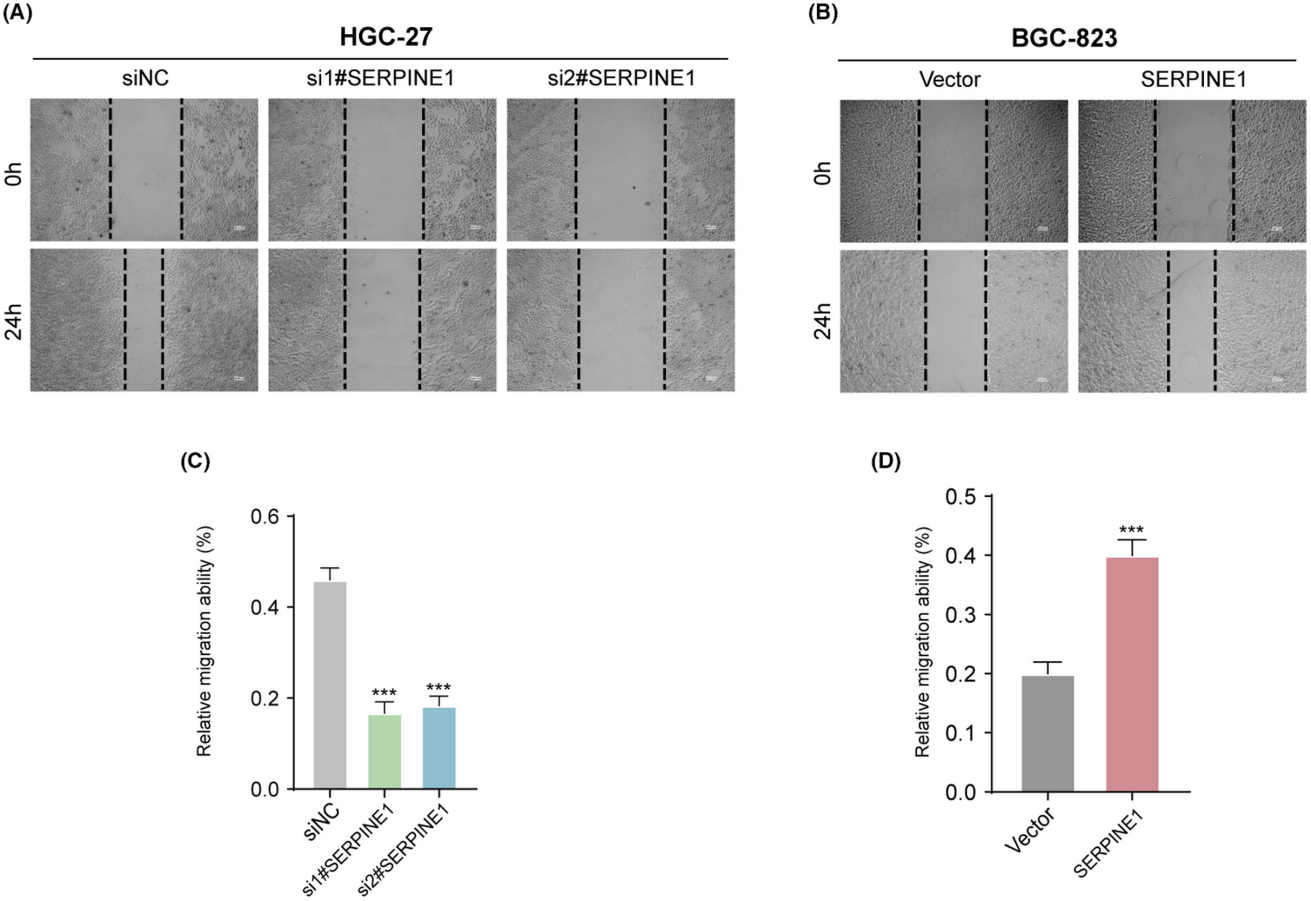
Impact of SERPINE1 on Gastric Cancer Cell Migration. (A, B) Evaluation of SERPINE1's influence on gastric cancer cell migration using a cell scratch assay. (C, D) Quantification of the migration distance difference between the two cell lines (***p < 0.001).

**FIGURE 10:**
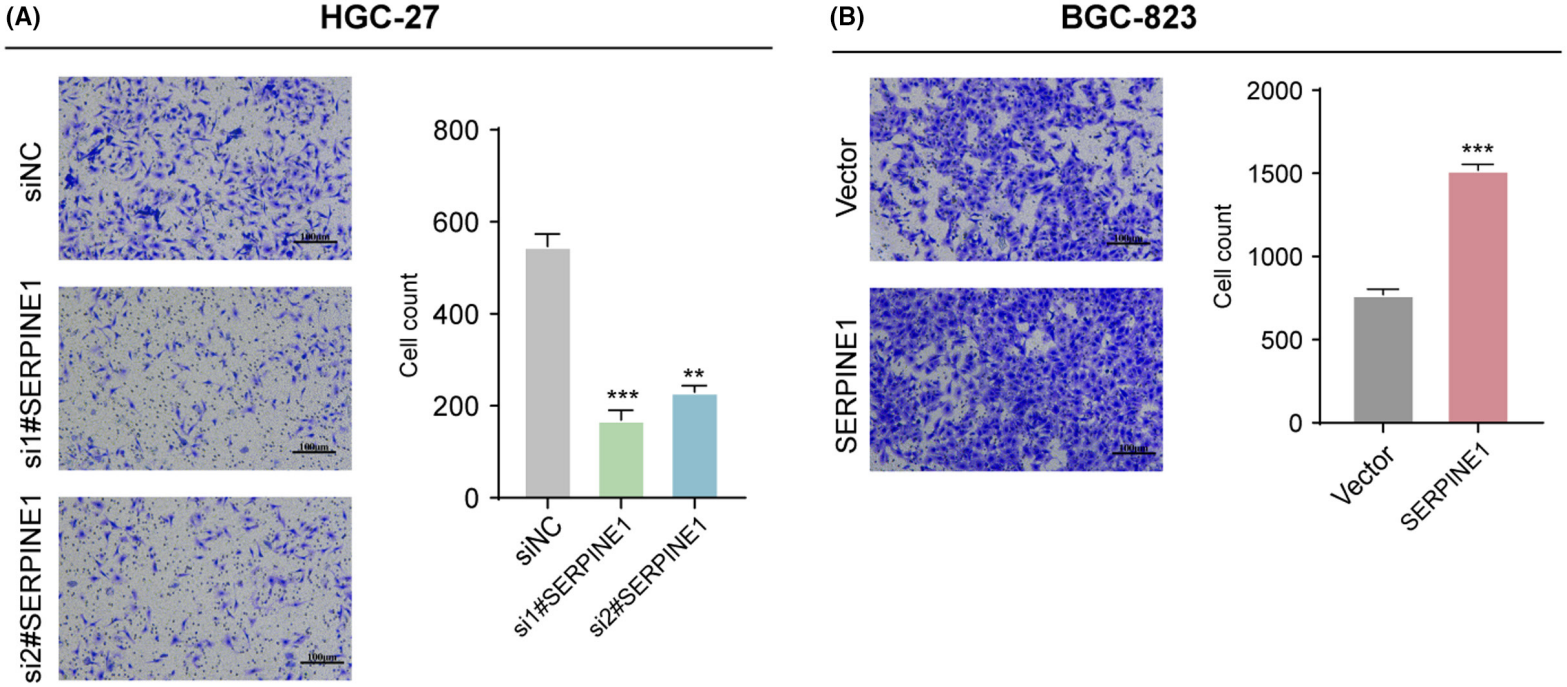
Effect of SERPINE1 on the migration of gastric cancer cells. (A) Knocking down SERPINE1 reduced the migration capacity of HGC‐27 cells; (B) Overexpression of SERPINE1 enhanced the migration ability of BGC‐823 cells (***p < 0.001).

The impact of SERPINE1 on the migratory activity of the GC cell lines HGC‐27 and BGC‐823 was investigated using wound healing assays. Following the knockdown of HGC‐27 cells with two different SERPINE1 siRNAs (si1#‐SERPINE1 and si2#‐SERPINE1), a significant reduction in wound closure ability was observed compared to the siNC group (Figure 9A,C), with the difference being highly statistically significant. Conversely, overexpression in BGC‐823 cells showed enhanced wound closure capability relative to the vector‐transfected control group (Vector), indicating that overexpression of SERPINE1 significantly promotes the migration of BGC‐823 cells (Figure 9B,D). These results consistently suggest that SERPINE1, by regulating the migratory capacity of GC cells, may play an important role in the aggressiveness and metastatic process of GC. The effect of SERPINE1 on the migration activity was further investigated by Transwell migtation assay. The experimental data shows that the number of cells migrating through Transwell chambers in the si1#‐SERPINE1 and si2#‐SERPINE1 groups was significantly reduced relative the siNC group, indicating that SERPINE1 silencing inhibited the migration ability of HGC‐27 cells (Figure 10A). Migration capacity of BGC‐823 after overexpression of SERPINE1. Compared with Vector, the SERPINE1‐overexpresses group had a significantly higher number of cell migrations across Transwell cells revealing a potential role for SERPIEN1 in promoting BGC‐823 cell migration (Figure 10B).

### 3.8 SERPINE1 increases GC cell proliferation

**FIGURE 11:**
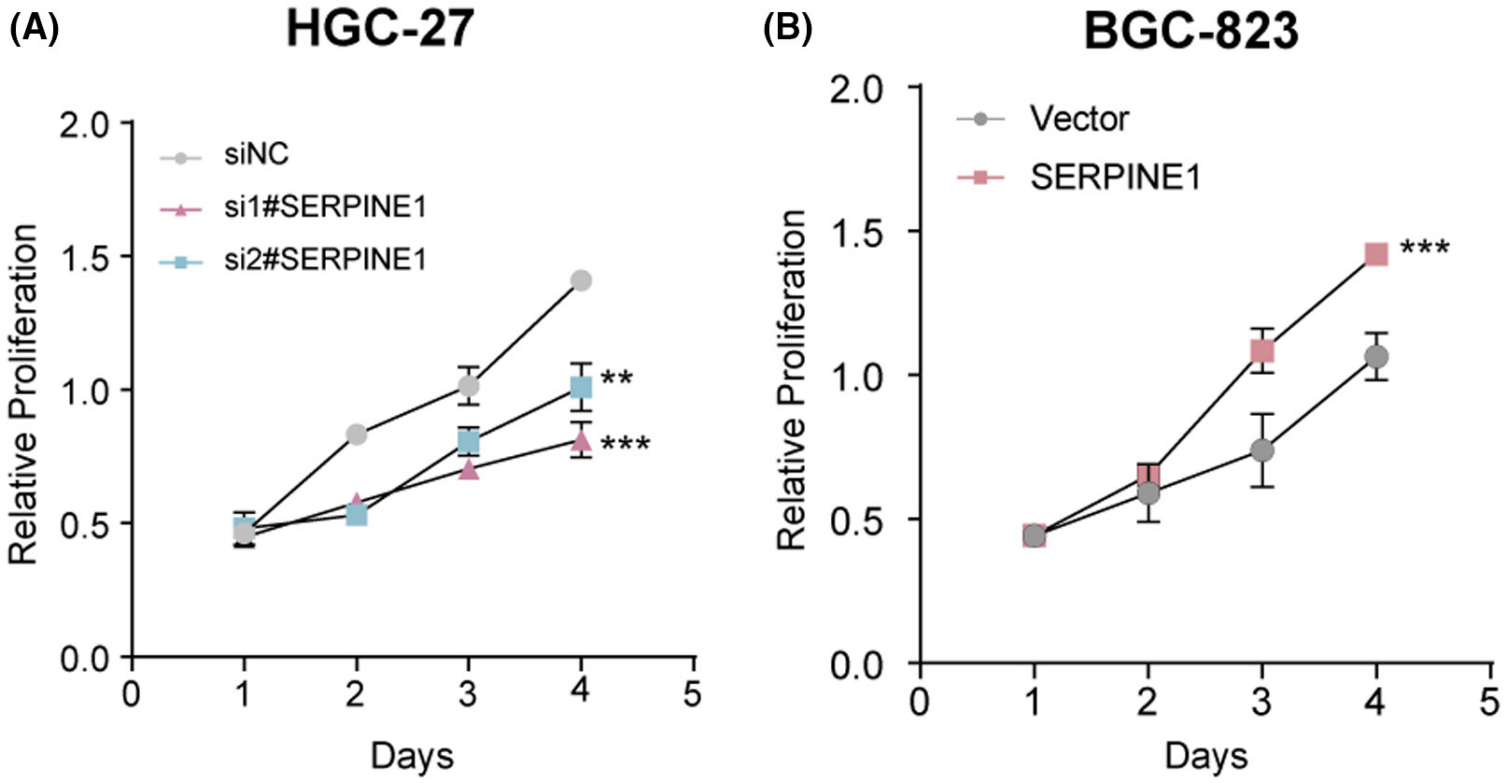
SERPINE1 changes the proliferation capacity of GC cells. (A) CCK‐8 assay showed inhibition of proliferation of GC cells. (B) The proliferation ability of GC cells was promoted by CCK‐8 assay(**p < 0.01, ***p < 0.001).

We further investigated the effect of SERPINE1 on the proliferative capacity of GC cells using the CCK‐8 proliferation assay. The CCK‐8 assay revealed that silencing with si#1SERPINE1 and si#2SERPINE1 resulted in significant proliferation inhibition compared to the nonspecific control (siNC) (Figure 11A). In the BGC‐823 cell line, overexpression of SERPINE1 showed a significant promotion of proliferation compared to the Vector (Figure 11B). These results suggest that SERPINE1 regulates the proliferation of GC cells, with its expression level being positively correlated with cell proliferative capacity.

## 4 DISCUSSION

The mortality rate from cancer has been showing an increasing trend worldwide and has now become a leading cause of death in many countries^17^ Surgical resection, radiation therapy, adjuvant chemotherapy, as well as immunotherapy and targeted therapy are the most common cancer treatment methods, but their efficacy is quite limited^18^ With the continuous development of bioinformatics, a vast accumulation of databases has been instrumental in aiding the search for tumour‐related biomarkers, molecular pathways and more.^19^ We explored the pan‐cancer expression profile of SERPINE1 and its aberrant expression in various cancers in relation to patient prognosis. Moreover, our study found a correlation between the expression levels of SERPINE1 and the prognosis of patients with GC, suggesting SERPINE1 as a potential biomarker related to GC prognosis. In this research, we utilized GC transcriptome data from the TCGA database, integrated with immune and stromal scores, to explore potential prognostic‐related genes. Specifically, we conducted an extensive analysis of the expression patterns of the SERPINE1 gene in various cancers and assessed its relationship with patient prognosis. The results support the potential of SERPINE1 as a biomarker related to the prognosis of patients with GC. These findings provide valuable insights for molecular diagnostics and therapeutic strategies for GC. The pan‐cancer analysis offers an important overview of the evolution of cancer prevention and treatment strategies. Through this analysis, it was found that SERPINE1 is differentially overexpressed in various tumours and significantly affects the prognosis of most cancers, including GC. With recent advances in molecular and targeted drug research, TMB has become a hotspot in tumour research, encompassing the tumour microenvironment, including surrounding blood vessels, immune cells, fibroblasts, extracellular matrix, signalling molecules and intercellular interactions.^20^, ^21^ Evaluating SERPINE1's prognostic role in GC, considering the immune cell infiltration, revealed a significant positive correlation between SERPINE1 and the infiltration of CD8+ T cells, macrophages, neutrophils and dendritic cells. The expression level in GC also showed a clear positive correlation with stromal and immune scores. These results may suggest that SERPINE1 plays a role in immune regulation in GC. To further investigate the relationship between SERPINE1 and the efficacy of immunotherapy, we studied the correlation between the expression of SERPINE1 and TMB and MSI. In GC, the expression of SERPINE1 was correlated with both TMB and MSI, which could provide clues for seeking new immunotherapy targets, but further clinical trials and substantiation are needed. Furthermore, we used cellular assays to explore the impact of SERPINE1 on the functional biology of GC cells. Using two GC cell lines, HGC‐27 and BGC‐823, the migration ability of both cell types was significantly affected by knocking down or overexpressing SERPINE1, as evidenced by wound healing and Transwell assays. CCK‐8 assay results showed that knocking down or overexpressing SERPINE1 significantly impacted the proliferation ability of both cell types. This study, through pan‐cancer analysis and other biological methods, combined with molecular biological experiments, investigated the role and mechanism of SERPINE1 in tumour development, discovering its potential as a prognostic marker in GC. Moreover, SERPINE1's correlation with the tumour immune microenvironment, microsatellite instability and tumour mutation burden was identified. This research offers new insights and directions for the clinical prognosis assessment and molecular target drug development in GC.

## 5 CONCLUSIONS

We undertook a comprehensive pan‐cancer analysis and a range of in vitro studies to deepen our understanding of the SERPINE1 gene. Aligning with findings from earlier research, SERPINE1 is notably upregulated in a majority of cancer types, suggesting its potential as a prognostic indicator across various tumours. Additionally, SERPINE1 is crucial in modulating the immune environment within the TME, particularly in the case of GC. Through GSEA, we discovered links between SERPINE1 and several biological pathways. In vitro tests further affirmed SERPINE1's role in the growth and advancement of GC. Hence, SERPINE1 could be an emerging marker gene for prognostic prediction and a potential therapeutic target in GC. Nonetheless, our research has certain limitations. Additional in‐depth functional mechanism studies and clinical trials are needed to corroborate our findings and gain a more comprehensive understanding of the SERPINE1 gene.

## AUTHOR CONTRIBUTIONS

**Yuming Ju:** Writing – original draft (equal). **Zeshen Wang:** Writing – review and editing (equal). **Qiancheng Wang:** Writing – review and editing (equal). **Shiyang Jin:** Validation (equal). **Pengcheng Sun:** Validation (equal). **Yuzhe Wei:** Validation (equal). **Guanyu Zhu:** Validation (equal). **Kuan Wang:** Supervision (equal).

## FUNDING INFORMATION

No funding was received for this article.

## CONFLICT OF INTEREST STATEMENT

The authors confirm that there are no conflicts of interest.

## Data Availability

The datasets presented in this study can be found in online repositories. The names of the repository/repositories and accession number(s) can be found in the article/Supplementary Material.
